# Occupational and Work‐Related Factors Associated With Job Esteem Among Nurses in Beijing: A Cross‐Sectional Study

**DOI:** 10.1002/nop2.70842

**Published:** 2026-09-30

**Authors:** Zixuan Yan, Xianzong Ma, Cunduo Jin, Huaguo Zhang, Qingqing Su, Meihua Ji, Wenyu Zhang

**Affiliations:** ^1^ School of Gerontological and Welfare China Civil Affairs University Beijing China; ^2^ Senior Department of Gastroenterology The First Medical Center of Chinese PLA General Hospital Beijing China; ^3^ Department of Nursing The Seventh Medical Center, Chinese PLA General Hospital Beijing China; ^4^ Beijing Key Laboratory of Intelligent Drug Research and Development for Mental Disorders, National Clinical Research Center for Mental Disorders, National Center for Mental Disorders Beijing Anding Hospital, Capital Medical University Beijing China; ^5^ Department of Nursing The First Medical Center of Chinese PLA General Hospital Beijing China; ^6^ School of Nursing Capital Medical University Beijing China

**Keywords:** intention to leave, job esteem, job satisfaction, nurses, working hours, workload

## Abstract

**Aim:**

To examine occupational and work‐related factors associated with job esteem among nurses in Beijing, with particular attention to workload, working‐time arrangements, job satisfaction, and intention to leave.

**Design:**

A multicenter cross‐sectional study.

**Methods:**

An anonymous online survey was completed by 1381 registered nurses from seven hospitals in Beijing between January 2023 and September 2024. Data were collected on demographic and occupational characteristics, working time, perceived workload, job satisfaction, intention to leave, and job esteem. A multivariable linear mixed‐effects model with a random intercept for hospital was used to examine factors associated with the total job esteem score.

**Results:**

Among the participants, 61.0% of the nurses worked 41 h or more per week, and 35.3% worked for at least 9 consecutive hours per day. The mean job esteem score was 81.8 ± 12.1, with professional authority and future value receiving the lowest dimension scores. Higher total job esteem scores were associated with a monthly income of ≥ RMB 10,001, contract or other employment relationships, and 6–20 years of working experience. Nurses working 41–45 h per week had lower scores than those working ≤ 40 h. Job satisfaction and intention to leave the current hospital showed the strongest associations with job esteem, although the estimates across job‐satisfaction categories did not follow a consistent stepwise pattern. Other working‐time and workload indicators were not independently associated with job esteem. Hospital tier was not independently associated with job esteem, and between‐hospital variation was negligible.

**Conclusions:**

Job esteem among nurses was associated with remuneration, employment relationships, work experience, weekly working hours, job satisfaction, and intention to leave. The findings support continued organizational attention to fair remuneration, early‐career support, professional recognition, transparent career‐development pathways, and reasonable working hours. Job satisfaction and intention to leave may also help organizations identify aspects of the work environment that require attention.

**Patient or Public Contribution:**

No patient or public contribution.

## 1 Introduction

Nurses continue to work in healthcare systems marked by complex and fluctuating patient‐care demands, persistent staffing shortages, and substantial physical and emotional burdens (Hanson et al. 2024; Li et al. 2024; Ware et al. 2024). In China, COVID‐19 temporarily added infection‐control procedures, patient screening, isolation requirements, and restrictions on hospital access to nurses' existing clinical responsibilities (Chen et al. 2021). Nurses involved in COVID‐19‐related medical work also experienced increased workloads, emotional strain, and changes in how their professional contribution was perceived by healthcare organizations and the public (Dittborn et al. 2022; Gualandi et al. 2023). Although these experiences provide relevant context for the present study, demanding workloads, insufficient organizational support, and concerns about professional recognition are not confined to the pandemic. They remain important issues for nursing workforce well‐being and retention.

Job esteem, conceptualized as the respect, value, and pride individuals associate with their occupational role, is especially relevant in nursing, a profession deeply rooted in principles of care and ethics (Yu et al. 2023). Drawing from social identity theory and self‐determination theory (Montoya and Pinter 2024; Ntoumanis et al. 2021), job esteem influences self‐perception, professional behaviours, and clinical performance (Dincer and Inangil 2021). Higher job esteem is associated with enhanced decision‐making, moral agency, and care quality (Ghaleh et al. 2024; Yu et al. 2023), while lower job esteem correlates with burnout, emotional detachment, and turnover intention (Cai et al. 2020; Liu et al. 2021; Wang et al. 2023). Nurses with strong job esteem demonstrate greater resilience when confronting ethical challenges, such as defending patient autonomy or addressing institutional unfairness (Cai et al. 2020). Conversely, diminished job esteem correlates with emotional exhaustion, depersonalization, and reduced accomplishment—core components of burnout (Liu et al. 2021). It may also foster frustration and disillusionment, ultimately predisposing nurses to leave the profession (Wang et al. 2023). Although job esteem is increasingly recognized as relevant to nurses' occupational well‐being and professional practice, empirical research on its associated factors remains limited. Existing nursing studies have focused primarily on burnout, psychological distress, job satisfaction, and turnover intention, with less attention paid to whether work intensity and job satisfaction are independently associated with job esteem. Nurses' previous COVID‐19 related medical work provides relevant occupational context, as such work may have involved increased demands and emotional strain (Kong et al. 2023; Zhang et al. 2021); however, their current job esteem may also reflect broader and continuing workplace conditions rather than pandemic‐specific experiences. In particular, sustained work demands, administrative and invisible work, organizational support, and professional recognition may contribute to how nurses evaluate their work and the value attached to their occupational role (Alahiane et al. 2023; Frennert et al. 2023; Huang et al. 2025). Therefore, examining the associations of work intensity and job satisfaction with job esteem may provide a more specific understanding of nurses' current occupational experiences.

Work intensity, defined as the effort expended by employees to fulfil organizational demands within constrained timeframes, encompasses dimensions such as working hours, task complexity, emotional labor, and cognitive load (Dincer and Inangil 2021; Liu et al. 2021). It has gained renewed attention as a critical factor affecting health workers, particularly during the COVID‐19 pandemic. Nurses faced unprecedented clinical demands, including high patient mortality, increased acuity, and the logistical and emotional burdens of implementing infection control measures, often while wearing restrictive personal protective equipment for extended periods in isolated settings (Dincer and Inangil 2021; Ghaleh et al. 2024). A study has reported that nurses exhibited higher anxiety than other health professionals during the pandemic, with uncontrolled work intensity being associated with psychological distress, burnout, and clinical errors (Cai et al. 2020). Excessive workload can diminish job satisfaction by constraining autonomy and work–life balance, thereby potentially reducing job esteem (Liu et al. 2021; Wang et al. 2023). Conversely, nurses who perceive societal appreciation and professional respect may be better able to withstand work intensity and find purpose in adversity (Zhang et al. 2021). However, the “hero narrative” prevalent during the pandemic, while momentarily uplifting, often lacked institutional support, creating a dissonance between symbolic recognition and unsustainable working conditions (Kong et al. 2023). Although previous studies have reported associations between work intensity, job satisfaction, and related occupational outcomes (Liu et al. 2021; Wang et al. 2023; Zhang et al. 2021), to our knowledge, few studies have examined work intensity, job satisfaction, and job esteem simultaneously. Consequently, it remains unclear whether work intensity and job satisfaction are independently associated with job esteem among nurses in China, particularly in the context of sustained healthcare demands.

The present study sought to address this gap by examining the relationships between work intensity, job satisfaction, and job esteem among nurses in Beijing. Specifically, we aimed to examine the independent associations of work intensity and job satisfaction with job esteem after accounting for other occupational variables. The findings may provide insights for healthcare administrators and policymakers seeking to improve nursing workforce stability and well‐being through evidence‐informed interventions.

## 2 Methods

### 2.1 Study Design, Setting, and Participants

This cross‐sectional study was conducted between January 2023 and September 2024 in seven hospitals in Beijing, China, including three tertiary hospitals, two secondary hospitals, and two primary hospitals. Eligible participants were registered nurses working in clinical nursing roles at the participating hospitals during the study period. The survey was coordinated with the assistance of a designated liaison in the nursing department of each hospital and administered using an online questionnaire.

Nurses were eligible to participate if they: (1) held a valid Chinese nursing licence, (2) were currently employed in a clinical nursing role, (3) had at least 2 years of full‐time clinical nursing experience, and (4) agreed voluntarily to participate. Nurses working exclusively in administrative or other non‐clinical positions were not eligible. Nurses who self‐reported a diagnosed psychiatric disorder or severe chronic condition that could substantially interfere with work or questionnaire completion were also excluded. This health‐related criterion was included as a precaution against imposing an additional research burden on nurses with serious health conditions; it was not based on temporary occupational stress or psychological symptoms that did not meet the stated diagnosis‐based criterion. Hospital nursing managers and liaisons used only the non‐sensitive employment information needed to prepare the initial sampling rosters. They did not apply the health‐related criterion or have access to nurses' diagnoses, medical records, self‐screening responses, or individual eligibility results. The health‐related criterion was assessed privately by the selected nurses through the online platform before they entered the main questionnaire. No diagnosis or supporting medical documentation was requested. This study formed part of a broader research project examining the professional development and working conditions of the nursing workforce in Beijing.

Ethical approval was obtained from the Committee for Ethics of Capital Medical University prior to study commencement (approval no. Z2023SY051). All participants received information about the study and provided electronic informed consent before eligibility confirmation and questionnaire completion. Participation was voluntary, and declining or discontinuing participation had no effect on employment, performance appraisal, promotion, or other workplace interests. Survey responses were collected without direct personal identifiers.

### 2.2 Questionnaire Development

The questionnaire was reviewed by a panel of five experts in nursing management and nursing research. Three rounds of review were conducted to improve the relevance, clarity, and appropriateness of the items. The final questionnaire contained three sections.

The first section collected demographic and employment information, including sex, age, marital status, average monthly income, professional title, employment type, and years of nursing experience.

The second section assessed work patterns, perceived workload, job satisfaction, and intention to leave. The work‐related items were developed and refined by the expert panel over three rounds of review. The items included: “Weekly working hours,” with responses categorized as ≤ 40 h, 41–45 h, 46–50 h, 51–55 h, 56–60 h, and ≥ 61 h; “Working days per week,” with options of ≤ 5, 6, or 7 days; “Night shifts per week,” categorized as none, 1–2, 3–4, or ≥ 5; “Do you have to take on any additional work outside of your duties” with responses of yes or no; “Perceived level of workload,” indicated on a Likert scale from 1 (a little) to 4 (extremely); “Frequency of working hours over 12 per day within a month,” with answers including never, 1–3 times/week, or ≥ 4 times/week; “Continuous working hours per day” categorized as ≤ 4 h, 5–8 h, 9–12 h, or ≥ 13 h; “How satisfied are you with your job” with options for very satisfied, a little satisfied, neutral, a little dissatisfied, and very dissatisfied; and “Would you leave your current hospital soon?” with responses of yes, not decide and no.

The third section assessed job esteem using the validated Chinese version of the Job‐Esteem Scale (JES). The original Korean‐language instrument was developed by Choi and Jung (2020), and the Chinese version was translated and culturally adapted by Shi and Shen (2021). The adaptation process included forward translation, back‐translation, expert consultation, cross‐cultural semantic equivalence assessment, and pilot testing. In their validation study involving 252 nurses from two tertiary hospitals in China, Shi and Shen (2021) reported good psychometric properties for the Chinese version, including a Cronbach's *α* of 0.942, test–retest reliability of 0.875, and a scale‐level content validity index (S‐CVI/Ave) of 0.98. Exploratory factor analysis supported a five‐factor structure that explained 69.87% of the total variance, and confirmatory factor analysis indicated acceptable model fit. This validated Chinese version has subsequently been used in studies of Chinese nurses, including the study by Guo et al. (2026), which reported a Cronbach's *α* of 0.970. The Chinese JES consists of 25 items across five dimensions: professional self‐awareness (4 items), social trust and respect (3 items), organizational respect and recognition (4 items), professional authority and future value (4 items), and professional competence (10 items). Each item was rated on a 5‐point Likert scale, with options ranging from “1 = completely disagree” to “5 = completely agree”. Total scores range from 25 to 125, with higher scores indicating greater job esteem. In the present study, Cronbach's *α* was 0.921 for the total scale. The Cronbach's *α* coefficients for the five dimensions were 0.890 for professional self‐awareness, 0.888 for social trust and respect, 0.877 for organizational respect and recognition, 0.940 for professional authority and future value, and 0.960 for professional competence.

### 2.3 Sampling and Data Collection

Systematic random sampling was conducted separately within each hospital. First, the nursing department at each hospital prepared a roster based solely on the non‐sensitive employment‐related eligibility criteria, including valid licensure, current clinical employment, and the required duration of clinical experience. Each potentially eligible nurse was assigned a study identifier, and a de‐identified roster containing these identifiers was provided to the research team. The research team did not receive names of nurses, telephone numbers, WeChat accounts, or other personal contact information.

A computer‐generated random number was used to determine the starting point on each hospital roster, after which identifiers were selected at a fixed interval according to the hospital‐specific sampling sequence. The selected identifiers were returned to a designated hospital liaison, who retained the identifier‐to‐contact linkage and contacted the corresponding nurses directly. Institutional email, direct messages through work‐related WeChat, and hospital internal messaging systems were used only to deliver study information to the selected nurses. The questionnaire link was not posted as an unrestricted public survey or distributed to nurses who had not been selected. Thus, systematic sampling determined who was approached, whereas the institutional communication channels were used to contact those selected.

Data collection was conducted using the SO JUMP platform (https://www.wjx.cn/). After opening the study page, selected nurses read the participant information and provided electronic informed consent. They then completed the confidential eligibility check. Nurses who confirmed that they met all eligibility criteria could proceed to the main questionnaire. Those who did not meet the criteria were not asked to disclose the relevant diagnosis or reason for ineligibility. Individual eligibility responses were not returned to hospital managers or liaisons and were not included in the analytical dataset. Recruitment followed the prespecified sampling sequence at each hospital until the planned sample size had been reached. Where a selected nurse did not meet all eligibility criteria, the next nurse in the sampling sequence was approached. The final invitation denominator therefore comprised nurses who had completed the confidential eligibility check and confirmed that they met all study criteria, rather than all nurses on the initial rosters or all nurses approached for eligibility confirmation. The platform was configured to restrict repeat submissions, and the main questionnaire did not collect directly identifying information. During the data‐collection period, hospital liaisons sent two general reminders to the selected sample. The reminders were not based on individual completion status, and the liaisons could not access or link individual nurses to their eligibility responses or questionnaire answers.

The sample size was determined using a precision‐based calculation for estimating a population mean on the item‐score metric, rather than an a priori power calculation for the multivariable regression model. The required sample size was calculated using the formula *N* = (*Uασ*/*δ*)^2^, where *Uα* = 1.96 corresponded to a two‐sided 95% confidence level, *σ* was the assumed standard deviation, and *δ* was the desired absolute precision (i.e., the confidence‐interval half‐width). Based on a standard deviation of 1.15 and an absolute precision of 0.066, the calculation yielded a minimum sample size of approximately 1166 nurses (Zhang et al. 2021, 2025). After adjustment for an anticipated 10% non‐response or incomplete‐response rate, the recruitment target was approximately 1296 nurses. The final analytic sample comprised 1381 nurses.

### 2.4 Data Management and Quality Control

Data collected through the online questionnaire platform were exported directly to an electronic database, thereby avoiding errors associated with manual data entry. Before analysis, submitted questionnaires were assessed against prespecified criteria for completeness, duplicate submission, out‐of‐range values, and logical inconsistency. Questionnaires failing any of these checks were to be excluded from the analytical sample. Because the predictor and outcome variables were collected through the same questionnaire, several procedural safeguards were implemented to reduce the risk of common method bias, including anonymous response collection, neutral instructions, and the placement of predictor and outcome measures in separate sections of the questionnaire. These procedural safeguards were intended to mitigate, rather than eliminate, the potential for common method bias.

### 2.5 Statistical Analysis

All analyses were performed using IBM SPSS Statistics for Windows, version 26.0 (IBM Corp., Armonk, NY, USA), and R, version 4.6.1. Categorical variables were summarized as frequencies and percentages, whereas continuous variables were described using means and standard deviations (SDs). The internal consistency of the questionnaire was evaluated using Cronbach's alpha. Because no validated cut‐off value has been established for the Job Esteem Scale, its total score (range: 25–125) was treated as a continuous outcome.

Given the hierarchical structure of the data, with nurses nested within hospitals, a linear mixed‐effects model was fitted to investigate factors associated with the total job esteem score. A hospital‐specific random intercept was included to account for potential within‐hospital correlation and unmeasured heterogeneity across hospitals. Hospital tier (primary, secondary, or tertiary) was modelled as a fixed effect. Nurse‐level demographic and work‐related characteristics, job satisfaction, and intention to leave were entered simultaneously as fixed effects. Categories with small numbers of participants were combined with conceptually similar categories before analysis (monthly income of ≤ RMB 3500 and RMB 3501–5000 were combined as ≤ RMB 5000; vice professor and chief nurse were combined as vice professor/chief nurse; contract employment and other labour relationships were combined as contract/other labour relationships; and six and seven working days per week were combined as 6–7 working days per week). All predictors were specified as categorical variables, with the lowest coded category used as the reference group.

The model was estimated using restricted maximum likelihood. Degrees of freedom for fixed‐effect tests were calculated using the Satterthwaite approximation. Fixed‐effect estimates were reported as regression coefficients (B) with standard errors (SEs), *t* statistics, 95% Wald confidence intervals (CIs), and two‐sided *p*‐values. Each coefficient represents the adjusted mean difference in the total JES score between the specified category and its reference category. Overall associations for categorical predictors were evaluated using omnibus *F*‐tests. Variance components for the hospital‐specific random intercept and residual error were estimated, and the intraclass correlation coefficient (ICC) was calculated to quantify the proportion of total variance attributable to between‐hospital differences. Singular fit was assessed using lme4. Marginal and conditional *R* ^*2*^ values were calculated to quantify the variance explained by the fixed effects and by the combined fixed and random effects, respectively. Model assumptions were assessed by examining residual‐versus‐fitted plots for homoscedasticity and Q–Q plots for residual normality. Multicollinearity among the fixed‐effect predictors was assessed using variance inflation factors (VIFs). The linear mixed‐effects model was implemented in R using the lme4, lmerTest, performance, and MuMIn packages. Statistical significance was defined as a two‐sided *p*‐value < 0.05.

## 3 Results

### 3.1 Participant Characteristics and Work Patterns

A total of 1394 nurses who had confirmed that they met all eligibility criteria were invited to complete the main questionnaire. Of these, 1381 completed and submitted the questionnaire, whereas 13 did not complete it. All submitted questionnaires met the prespecified quality checks and were retained for analysis. The response rate among eligible invited nurses was therefore 99.1% (1381/1394).

Most participants were female (95.4%), and 63.6% were aged ≤ 35 years. A total of 72.0% reported a monthly income exceeding RMB 8000 (approximately US$1127), whereas 3.6% held senior professional titles. In addition, 70.8% of the participants were married, 66.6% were contract nurses, and 5.1% had ≥ 31 years of nursing experience. Regarding work patterns, 42.8% of the nurses worked 41–45 h per week, and 88.5% worked five or fewer days per week. A total of 43.2% reported one or two night shifts per week, whereas 38.8% reported no night shifts. In addition, 35.3% worked continuously for at least 9 h per day, and 46.1% reported undertaking work outside their duties and responsibilities (Table 1).

**TABLE 1:** Characteristics of demographic, job satisfaction, working intensity of nurses in Beijing (*N* = 1381).

Variables	Frequency, *n* (%)
Gender	
Female	1317 (95.4)
Male	64 (4.6)
Age (years)	
≤ 35	879 (63.6)
≥ 36	502 (36.4)
Marital status	
Unmarried	374 (27.1)
Married	978 (70.8)
Divorce and others	29 (2.1)
Average monthly income (RMB)	
≤ 3500	62 (4.5)
3501–5000	90 (6.5)
5001–8000	235 (17.0)
8001–10,000	548 (39.7)
≥ 10,001	446 (32.3)
Positional title	
Nurse	288 (20.9)
Nurse practitioner	587 (42.5)
Nurse‐in‐charge	456 (33.0)
Vice professor	44 (3.2)
Chief nurse	6 (0.4)
Labor relationship	
Permanent nurse	437 (31.6)
Contract nurse	919 (66.6)
Others	25 (1.8)
Working experience	
1–5	306 (22.2)
6–10	287 (20.8)
11–15	379 (27.4)
16–20	193 (14.0)
21–25	92 (6.7)
26–30	54 (3.9)
≥ 31	70 (5.1)
Working intensity	
Weekly working hours	
≤ 40	538 (39.0)
41–45	591 (42.8)
46–50	159 (11.5)
51–55	39 (2.8)
56–60	22 (1.6)
≥ 61	32 (2.3)
Working days per week	
≤ 5	1222 (88.5)
6	152 (11.0)
7	7 (0.5)
Night shifts per week	
None	536 (38.8)
1–2	596 (43.2)
3–4	225 (16.3)
≥ 5	24 (1.7)
Do you have to take on any additional work outside of your duties?	
Yes	637 (46.1)
No	744 (53.9)
Perceived level of workload	
A little	81 (5.9)
Moderately	858 (62.1)
Quite	407 (29.5)
Extremely	35 (2.5)
Frequency of working hours > 12 per day within a month	
Never	737 (53.4)
1–3/week	572 (41.4)
≥ 4/week	72 (5.2)
Continuous working hours per day	
≤ 4	151 (10.9)
5–8	742 (53.7)
9–12	452 (32.7)
≥ 13	36 (2.6)
Job satisfaction	
Very satisfied	352 (25.5)
A little satisfied	509 (36.9)
Neutral	444 (32.2)
A little dissatisfied	53 (3.8)
Very dissatisfied	23 (1.7)
Whether you would leave your current hospital recently	
Yes	40 (2.9)
No	1063 (77.0)
Undecided	278 (20.1)

*Note:* RMB denotes Renminbi. For international comparison, approximate US dollar equivalents were calculated using an exchange rate of RMB 7.10 to US$1.00. These conversions are provided for interpretive purposes only; income was collected and analysed in RMB.

### 3.2 Job Esteem and Job Satisfaction Among Nurses

Overall, 62.4% of the participants reported being very satisfied or a little satisfied with their jobs, whereas 5.5% reported being a little dissatisfied or very dissatisfied. Regarding intention to leave their current hospital, 2.9% answered “yes,” 77.0% answered “no,” and 20.1% were undecided (Table 1).

The mean total job esteem scale score was 81.8 ± 12.1. As the subscales contained different numbers of items, their relative levels were compared using mean item scores rather than total subscale scores. Professional self‐awareness and social trust and respect had the highest mean item scores (both 3.4), followed by professional competence (3.3) and organizational respect and recognition (3.2). Professional authority and future value had the lowest mean item score (2.9). The corresponding total subscale scores were 13.7 ± 2.1 for professional self‐awareness, 10.3 ± 1.7 for social trust and respect, 12.8 ± 2.5 for organizational respect and recognition, 11.7 ± 2.9 for professional authority and future value, and 33.3 ± 5.0 for professional competence (Table 2).

**TABLE 2:** Characteristics of job‐esteem of nurses in Beijing (*N* = 1381).

Variable	Mean item score, mean ± SD	Total score, mean ± SD
Professional self‐awareness	3.4 ± 0.5	13.7 ± 2.1
Social trust and respect	3.4 ± 0.6	10.3 ± 1.7
Organizational respect and recognition	3.2 ± 0.6	12.8 ± 2.5
Professional authority and future value	2.9 ± 0.7	11.7 ± 2.9
Professional competence	3.3 ± 0.5	33.3 ± 5.0
Total job esteem scale score		81.8 ± 12.1

### 3.3 Factors Associated With Job Esteem Among Nurses in Beijing: Linear Mixed‐Model Analysis

A linear mixed model was used to examine factors associated with the total job esteem scale score, with hospital center included as a random intercept (Table 3). Hospital tier was not independently associated with the total job esteem scale score after adjustment for the other variables (*F* = 0.729, *p* = 0.482). The estimated hospital‐center random‐intercept variance was 0.000 (SD = 0.000), whereas the residual variance was 96.769 (SD = 9.837), yielding an ICC of 0.0000. The model was identified by lme4 as a singular fit because the random‐intercept variance was estimated at the boundary of zero. The marginal and conditional *R* ^*2*^ values were both 0.3542, indicating that the fixed effects explained approximately 35.4% of the variance, with no additional variance explained by the hospital‐center random effect. Collinearity diagnostics indicated an elevated VIF for years of working experience (12.64), likely reflecting its close relationships with age and positional title. The VIFs for age and positional title were 3.64 and 4.57, respectively, whereas those for all remaining predictors, including monthly income, were below 2.1. Accordingly, the coefficients for working experience should be interpreted cautiously.

**TABLE 3:** Linear Mixed Model of factors associated with the total job esteem scale score among nurses in Beijing (*N* = 1381).

Variable	B (95% Wald CI)	SE	*t*	*p*
Intercept	90.319 (86.726 to 93.912)	1.833	49.273	< 0.001
Hospital tier				
Primary hospital	Reference			
Secondary hospital	−0.466 (−2.508 to 1.576)	1.042	−0.447	0.655
Tertiary hospital	0.255 (−1.693 to 2.203)	0.994	0.256	0.798
Gender				
Female	Reference			
Male	−0.205 (−2.763 to 2.354)	1.305	−0.157	0.876
Age (years)				
≤ 35	Reference			
≥ 36	−0.011 (−2.070 to 2.048)	1.051	−0.010	0.992
Marital status				
Unmarried	Reference			
Married	−0.456 (−2.082 to 1.171)	0.830	−0.549	0.583
Divorce and others	0.357 (−3.596 to 4.310)	2.017	0.177	0.859
Average monthly income (RMB)				
≤ 5000	Reference			
5001–8000	−0.822 (−3.052 to 1.408)	1.138	−0.723	0.470
8001–10,000	0.750 (−1.285 to 2.786)	1.038	0.723	0.470
≥ 10,001	2.464 (0.304 to 4.624)	1.102	2.235	0.026
Positional title				
Nurse	Reference			
Nurse practitioner	−1.381 (−3.365 to 0.603)	1.012	−1.364	0.173
Nurse‐in‐charge	−0.948 (−3.333 to 1.436)	1.217	−0.780	0.436
Vice professor or chief nurse	0.038 (−3.824 to 3.899)	1.970	0.019	0.985
Labor relationship				
Permanent nurse	Reference			
Contract nurse and others	1.921 (0.421 to 3.422)	0.765	2.510	0.012
Working experience				
1–5	Reference			
6–10	3.356 (1.198 to 5.514)	1.101	3.048	0.002
11–15	2.667 (0.273 to 5.061)	1.221	2.184	0.029
16–20	3.567 (0.374 to 6.761)	1.629	2.190	0.029
21–25	3.262 (−0.439 to 6.964)	1.889	1.727	0.084
26–30	−0.134 (−4.193 to 3.924)	2.071	−0.065	0.948
≥ 31	2.392 (−1.642 to 6.426)	2.058	1.162	0.245
Working intensity				
Weekly working hours				
≤ 40	Reference			
41–45	−1.542 (−2.762 to −0.321)	0.623	−2.476	0.013
46–50	−1.166 (−3.076 to 0.745)	0.975	−1.196	0.232
51–55	−0.819 (−4.157 to 2.519)	1.703	−0.481	0.631
56–60	−2.073 (−6.592 to 2.447)	2.306	−0.899	0.369
≥ 61	−0.112 (−4.009 to 3.785)	1.989	−0.056	0.955
Working days per week				
≤ 5	Reference			
≥ 6	−0.088 (−1.864 to 1.689)	0.906	−0.097	0.923
Night shifts per week				
None	Reference			
1–2	−0.414 (−1.792 to 0.964)	0.703	−0.589	0.556
3–4	−0.747 (−2.542 to 1.048)	0.916	−0.816	0.415
≥ 5	1.103 (−3.112 to 5.318)	2.151	0.513	0.608
Do you have to take on any additional work outside of your duties?				
No	Reference			
Yes	−0.468 (−1.630 to 0.695)	0.593	−0.788	0.431
Perceived level of workload				
A little	Reference			
Moderately	−1.823 (−4.182 to 0.536)	1.204	−1.515	0.130
Quite	−2.538 (−5.113 to 0.038)	1.314	−1.931	0.054
Extremely	−1.428 (−5.853 to 2.998)	2.258	−0.632	0.527
Frequency of working hours > 12 per day within a month				
Never	Reference			
1–3/week	0.277 (−0.995 to 1.549)	0.649	0.427	0.669
≥ 4/week	−1.059 (−3.854 to 1.736)	1.426	−0.743	0.458
Continuous working hours per day				
≤ 4	Reference			
5–8	0.853 (−0.926 to 2.632)	0.908	0.940	0.347
9–12	1.815 (−0.180 to 3.809)	1.018	1.783	0.075
≥ 13	2.021 (−2.030 to 6.072)	2.067	0.978	0.328
Job satisfaction				
Very satisfied	Reference			
A little satisfied	−8.899 (−10.317 to −7.482)	0.723	−12.305	< 0.001
Neutral	−14.004 (−15.607 to −12.402)	0.818	−17.129	< 0.001
A little dissatisfied	−19.077 (−22.188 to −15.966)	1.587	−12.020	< 0.001
Very dissatisfied	−17.133 (−21.821 to −12.444)	2.392	−7.162	< 0.001
Intention to leave				
No	Reference			
Yes	−6.108 (−9.444 to −2.773)	1.702	−3.589	< 0.001
Undecided	−3.860 (−5.363 to −2.357)	0.767	−5.034	< 0.001

*Note:* B represents the adjusted mean difference in the total job esteem scale score relative to the corresponding reference category. Positive coefficients indicate higher adjusted mean scores, whereas negative coefficients indicate lower adjusted mean scores. Hospital was included as a random intercept. For the regression analysis, the following categories from Table 1 were combined: income ≤ RMB 3500 and RMB 3501–5000 as ≤ RMB 5000; vice professor and chief nurse as vice professor/chief nurse; contract employment and other labour relationships as contract/other labour relationships; and six and seven working days per week as 6–7 days per week.

Abbreviations: CI, confidence interval; RMB, renminbi.

After adjustment for the other variables, nurses with an average monthly income of RMB ≥ 10,001 had a mean job esteem scale score 2.464 points higher than those earning RMB ≤ 5000 (*B* = 2.464, 95% Wald CI: 0.304 to 4.624, *p* = 0.026). Contract nurses and nurses with other labour relationships had a mean score 1.921 points higher than permanent nurses (*B* = 1.921, 95% Wald CI: 0.421 to 3.422, *p* = 0.012). Compared with nurses with 1–5 years of working experience, those with 6–10 years (*B* = 3.356, 95% Wald CI: 1.198 to 5.514, *p* = 0.002), 11–15 years (*B* = 2.667, 95% Wald CI: 0.273 to 5.061, *p* = 0.029), and 16–20 years (*B* = 3.567, 95% Wald CI: 0.374 to 6.761, *p* = 0.029) had significantly higher mean job esteem scale scores. The other categories of working experience were not significantly associated with the total score. Nurses working 41–45 h per week had a mean score 1.542 points lower than those working ≤ 40 h per week (*B* = −1.542, 95% Wald CI: −2.762 to −0.321, *p* = 0.013), whereas the other weekly working‐hour categories were not statistically significant. Job satisfaction was strongly associated with job esteem. Compared with nurses who were very satisfied with their jobs, the adjusted mean job esteem scale score was lower among those who were a little satisfied (*B* = −8.899, 95% Wald CI: −10.317 to −7.482, *p* < 0.001), neutral (*B* = −14.004, 95% Wald CI: −15.607 to −12.402, *p* < 0.001), a little dissatisfied (*B* = −19.077, 95% Wald CI: −22.188 to −15.966, *p* < 0.001), or very dissatisfied (*B* = −17.133, 95% Wald CI: −21.821 to −12.444, *p* < 0.001). Compared with nurses who did not intend to leave their current hospital, those who intended to leave had a significantly lower mean job esteem scale score (*B* = −6.108, 95% Wald CI: −9.444 to −2.773, *p* < 0.001). Nurses who were undecided about leaving also had a lower mean score (*B* = −3.860, 95% Wald CI: −5.363 to −2.357, *p* < 0.001).

Gender, age, marital status, positional title, working days per week, night‐shift frequency, additional work outside regular duties, perceived workload, frequency of working more than 12 h per day, and continuous working hours per day were not significantly associated with the total job esteem scale score in the adjusted model (all *p* > 0.05). The remaining categories of monthly income were also not statistically significant. Overall, the absence of an independent hospital‐tier effect, together with the zero estimated hospital‐centre variance, indicated that the observed variation in job esteem was primarily attributable to individual‐level rather than hospital‐level differences. Model diagnostics were assessed using residual‐versus‐fitted and Q–Q plots. The residual‐versus‐fitted plot showed no clear systematic pattern or evidence of substantial heteroscedasticity. The Q–Q plot indicated generally acceptable residual normality, although mild deviations were observed in the tails.

## 4 Discussion

This cross‐sectional study examined occupational and work‐related factors associated with job esteem among nurses in Beijing. In the adjusted linear mixed‐effects model accounting for the clustering of nurses within hospitals, higher total JES scores were associated with a monthly income of ≥ 10,001 RMB, contract or other employment relationships, and 6–20 years of working experience. Nurses working 41–45 h per week had lower total JES scores than those working ≤ 40 h per week. Job satisfaction and intention to leave the current hospital were also associated with job esteem. Hospital tier was not independently associated with the total JES score, and the estimated between‐hospital variation was negligible. By contrast, perceived workload, additional work outside assigned responsibilities, extended workdays, and continuous working hours were not independently associated with the total JES score after adjustment for the other variables in the model. The pattern of associations suggests that job esteem reflects more than exposure to demanding working conditions; it is also related to nurses' employment experience and their evaluation of their current work situation.

The demographic profile of the study sample revealed several notable characteristics. Female nurses accounted for 95.4% of the sample, which was consistent with the national sex distribution of nurses in China (97.0%) (Zhang et al. 2021). Such an imbalance warrants attention because it may influence workforce diversity and professional development. The relatively young age structure of the sample, with 63.6% of participants aged ≤ 35 years, differed from national nursing workforce data (Wang et al. 2023; Zhang et al. 2021). This difference may reflect recent recruitment patterns or the turnover of older nurses in urban healthcare settings and highlights the need for further research on career sustainability in high‐pressure healthcare environments. Contract nurses accounted for 66.6% of the sample, indicating that non‐permanent employment arrangements were common among the participating nurses. Such arrangements may affect job security, employment benefits, and job satisfaction. In addition, 72.0% of the participants reported an average monthly income of > 8000 RMB. This income distribution may reflect Beijing's relatively high cost of living, the concentration of tertiary hospitals, and regional differences in healthcare funding (Zhang et al. 2021).

Our findings showed a substantial level of work intensity among the participating nurses. A total of 61.0% worked more than 40 h per week, and 35.3% worked continuously for at least 9 h per day. These demanding working conditions may reflect multiple factors, including sustained pressures following the COVID‐19 pandemic, Beijing's role as a national medical center receiving a high volume of complex cases, and potentially inadequate nurse staffing. Of particular concern, 46.1% of nurses reported regularly undertaking tasks beyond their formally defined responsibilities, while 32.0% perceived their workload as excessive. These findings are consistent with previous evidence linking prolonged working hours in healthcare settings to adverse occupational outcomes, including an increased risk of burnout among those working more than 40 h per week, as well as the substantial psychological burden experienced by healthcare workers during the COVID‐19 pandemic (Pappa et al. 2020). The high proportion of nurses undertaking duties outside their formal roles may also indicate systemic problems, such as role ambiguity, unclear responsibility boundaries, or persistent understaffing, all of which warrant greater attention from nursing administrators.

The mean total job esteem scale score in our study was 81.8 ± 12.1. Among the dimensions of job esteem, professional authority and future value had the lowest mean item score (2.9) indicating that nurses perceived professional authority and future career value as the comparatively weakest aspect of their job esteem. This finding may reflect less favourable perceptions of the professional standing of nursing and its prospects for long‐term development. This is relevant because opportunities for professional development and advancement have been identified by nurses as important forms of professional recognition (Sweeney and Wiseman 2023). The relatively low score for organizational respect and recognition further indicates that recognition within the workplace is an important aspect of nurses' job esteem. Previous research has shown that nurses' experience of professional respect is closely related to interactions with other healthcare professionals, workplace characteristics, professional autonomy, and involvement in decision‐making (Stievano et al. 2018). These findings suggest that strengthening job esteem requires attention not only to nurses' individual competence, but also to how their professional contribution is recognized and how opportunities for future development are provided within the organization. Greater participation in clinical and organizational decision‐making, clearer career‐development pathways, and more visible recognition of nursing contributions may therefore be relevant areas for nursing management.

Monthly income was associated with job esteem, with the clearest difference observed among nurses earning ≥ RMB 10,001 per month, whose scores were higher than those earning ≤ RMB 5000. No significant differences were found for the intermediate income groups. This suggests that the association may be more apparent at the upper end of the income distribution rather than increasing consistently across income levels. Remuneration is likely to matter not only as financial compensation but also as a visible acknowledgement of nurses' knowledge, responsibilities, and contribution to patient care. A recent Chinese study similarly reported an association between personal monthly income and occupational happiness among nurses, although occupational happiness and job esteem represent different aspects of work‐related evaluation (Liu et al. 2025). Higher income may also accompany greater clinical experience, specialist expertise, or more demanding roles, which may partly account for the difference observed here.

Employment relationship was also associated with job esteem. Nurses employed on contract or under other employment arrangements reported higher scores than permanent nurses. The reason for this finding is not clear from our data. Employment status in Chinese hospitals is often linked with differences in salary structure, performance incentives, job mobility, role allocation, and opportunities for advancement, and these features may be more relevant to job esteem than contractual status itself. Recent evidence from Chinese nurses has also shown that employment type is related to perceived professional benefit (Chen, Liu, and Lai 2026; Chen, Lu, et al. 2026). Our result therefore draws attention to the broader employment conditions surrounding different contractual arrangements rather than to the contractual category alone.

Working experience was another factor associated with job esteem. Nurses with 6–20 years of experience had higher scores than those in their first 1–5 years of practice. The differences were not maintained across all experience groups, so the result appears to distinguish early‐career nurses from those in the middle stages of their careers rather than showing a steady rise in job esteem with seniority. The first few years of nursing practice often involve adjustment to clinical responsibility, workplace expectations, and the gap between professional expectations and everyday practice. Among junior nurses in China, greater reality shock has recently been associated with weaker professional identity (Li et al. 2026), and this may help explain why nurses in the earliest career stage evaluated their occupational worth less positively. With accumulated clinical experience, nurses may become more confident in their professional role and receive greater recognition from colleagues and patients. The lack of a corresponding difference among nurses with the longest experience, however, suggests that years in practice alone do not account for higher job esteem.

The findings for working time were more limited. Nurses working 41–45 h per week had lower job esteem scores than those working ≤ 40 h, whereas the other weekly working‐hour categories were not significantly different. Perceived workload, additional work outside regular duties, working more than 12 h per day, continuous working hours, working days per week, and night‐shift frequency were also unrelated to job esteem after adjustment for the other variables. Thus, the present results do not show a consistent relationship between greater workload or longer working time and lower job esteem. The isolated finding for 41–45 h may reflect aspects of scheduling or work organization that are not captured by total weekly hours. This distinction is worth noting because longer working hours and shift arrangements have been associated with other nursing outcomes, including turnover intention, occupational stress, and perceived professional benefit (Kong et al. 2026; Xie et al. 2026). In the present study, however, workload indicators were less closely related to job esteem than employment characteristics and nurses' evaluations of their current work.

The most pronounced adjusted differences in job esteem were seen across levels of job satisfaction. Compared with nurses who were very satisfied with their jobs, all other satisfaction groups had substantially lower scores. The size of these differences was greater than that observed for income, employment relationship, working experience, or weekly working hours. The pattern was not strictly monotonic, however. Compared with the very satisfied group, the adjusted coefficient was smaller in magnitude for nurses who were very dissatisfied (*B* = −17.0, *n* = 23) than for those who were a little dissatisfied (*B* = −19.0, *n* = 53). This difference should be interpreted with some caution because relatively few nurses were in the very dissatisfied group. This close association is understandable because both constructs concern how nurses evaluate their working lives, although they are not identical. Job satisfaction refers more directly to the experience of the current job, whereas job esteem concerns the value and worth attached to nursing work. Recent multicenter evidence from China has likewise shown close links between job satisfaction and professional identity among nurses (Chen, Liu, and Lai 2026; Chen, Lu, et al. 2026). The large coefficients observed in our study may therefore reflect both a meaningful association between the two constructs and their conceptual proximity.

Intention to leave the current hospital showed a similar relationship. Nurses who intended to leave had lower job esteem scores, and lower scores were also found among those who were undecided. Previous research has linked nurses' perceived professional benefits with lower turnover intention, including among junior registered nurses in China (Lu and He 2026), while national data have identified work satisfaction as an important correlate of turnover intention (Xie et al. 2026). An important distinction in our study is that participants were asked whether they intended to leave their current hospital, not whether they intended to leave nursing. The association may therefore reflect dissatisfaction with the present organizational setting as well as nurses' broader views of their profession. The lower job esteem found among undecided nurses is also noteworthy, as uncertainty about remaining in the hospital may emerge before a definite intention to leave is formed. As with job satisfaction, the magnitude of this association should be considered in light of the conceptual connection between intention to leave and nurses' evaluations of their work.

Overall, the adjusted analysis places greater emphasis on nurses' employment circumstances, career stage, and evaluation of their current job than on workload alone. Income, employment relationship, working experience, job satisfaction, and intention to leave were all associated with job esteem, whereas most indicators of workload and working‐time intensity were not. Neither hospital tier nor between‐hospital variation appeared to contribute substantially to differences in job esteem after adjustment for individual‐level factors. For nursing management, this suggests that efforts to strengthen job esteem may need to extend beyond workload reduction and include fair recognition of professional contribution, support during the early career period, opportunities for career development, and attention to aspects of the work environment that shape satisfaction and willingness to remain.

### 4.1 Strengths and Limitations

This study contributes to the limited evidence on nurses' job esteem in China following the COVID‐19 pandemic. Rather than examining job esteem in isolation, it considers this concept alongside job satisfaction, intention to leave, and several aspects of nurses' working conditions. The findings provide a broader picture of how nurses' professional perceptions relate to their everyday work experiences. In particular, the study highlights the associations of income, employment relationship, working experience, and weekly working hours with job esteem. It also draws attention to the close relationships among job esteem, job satisfaction, and nurses' intention to remain in their current hospital. Furthermore, the use of a linear mixed‐effects model accounted for the hierarchical structure of nurses nested within hospitals and enabled the assessment of both hospital tier and between‐hospital variation. These findings may help healthcare managers better understand the factors associated with how nurses value their profession and role.

Several limitations should be acknowledged. First, the cross‐sectional, self‐reported data preclude causal inference and remain susceptible to social desirability bias and common method variance despite procedural safeguards. Second, hospital‐based recruitment and voluntary participation may have introduced selection bias. Moreover, the restriction to seven hospitals in Beijing, together with the exclusion of nurses with diagnosed psychiatric disorders or severe chronic conditions, limits the generalizability of the findings and may have produced a comparatively healthier sample. Third, although the mixed‐effects model accounted for the clustering of nurses within hospitals, the small number of hospitals may have reduced the precision of the estimated hospital‐level variance and intraclass correlation coefficient. Fourth, although established translation procedures were followed, translation may not fully preserve the semantic and cultural equivalence of the original instrument. Further cross‐cultural validation is therefore warranted. Fifth, given the large number of fixed‐effect comparisons and the absence of adjustment for multiple testing, findings with *p*‐values close to 0.05 should be interpreted cautiously. Finally, data collection extended from January 2023 to September 2024, during which staffing and workload conditions may have changed. Although the model accounted for hospital clustering and several measured work‐related characteristics, it did not explicitly model calendar‐time effects; residual temporal variation therefore cannot be excluded. In addition, job satisfaction and intention to leave were assessed using expert‐panel‐developed single items rather than validated multi‐item instruments, which may have limited measurement reliability and construct coverage.

### 4.2 Implications for Nursing Practice, Management, and Policy

The findings suggest that improving nurses' job esteem requires attention to both individual support and employment conditions. Nurse leaders should provide channels for staff to express work‐related concerns and access appropriate professional or psychological support. Particular attention may be needed for nurses in the first 5 years of practice through mentoring, career guidance, and opportunities to develop clinical competence.

Hospital managers should regularly review remuneration, employment arrangements, working hours, and career‐development opportunities. Salary and incentive systems should reflect nurses' qualifications, responsibilities, and clinical contributions. Transparent promotion pathways, professional recognition, and greater involvement of nurses in clinical and organizational decision‐making may help strengthen professional authority and perceived future value. Job satisfaction and intention to leave may also be monitored alongside job esteem to identify nurses who need further organisational support.

At the policy level, workforce strategies should support fair remuneration, reasonable working hours, and equitable career advancement across employment arrangements. Although most workload indicators were not independently associated with job esteem in this study, appropriate staffing and adequate recovery time remain important for nurses' well‐being and quality of care. As causal effects cannot be inferred from the present findings, the effects of these strategies on job esteem require further evaluation in longitudinal or intervention studies.

## 5 Conclusions

Job esteem among nurses was associated with several occupational and work‐related factors. Higher scores were found among nurses earning ≥ RMB 10,001 per month, those with contract or other employment relationships, and those with 6–20 years of work experience. Nurses working 41–45 h per week had lower scores than those working ≤ 40 h, while other indicators of working hours and workload were not significant in the adjusted model. Job satisfaction and intention to leave the current hospital showed the strongest associations with job esteem, although the estimates across job‐satisfaction categories did not follow a consistent stepwise pattern. Hospital tier was not independently associated with job esteem, and there was little evidence of between‐hospital variation in this sample. The relatively low scores for professional authority and future value further suggest concerns about nurses' professional standing and career development.

These findings support continued attention to fair remuneration, early‐career support, professional recognition, transparent career‐development pathways, and reasonable working hours. Job satisfaction and intention to leave may also help organizations identify aspects of the work environment that require attention. However, their close links with job esteem should be kept in mind when interpreting the strength of these associations. Given the cross‐sectional design, no causal conclusions can be drawn. The study was also limited to seven hospitals in Beijing, and the small number of hospitals restricts the interpretation of hospital‐level variation and the generalizability of the findings. Longitudinal studies involving a wider range of institutions and regions are needed to examine how job esteem develops over time and whether organizational changes can strengthen it.

## Author Contributions

**Zixuan Yan:** methodology, investigation, writing – original draft, data curation, resources. **Xianzong Ma:** investigation, methodology, supervision, writing – original draft, writing – review and editing. **Cunduo Jin:** data curation, investigation, writing – review and editing. **Huaguo Zhang:** data curation, investigation. **Qingqing Su:** data curation, investigation. **Meihua Ji:** investigation, data curation. **Wenyu Zhang:** conceptualization, methodology, software, data curation, supervision, resources, project administration, formal analysis, validation, visualization, writing – review and editing, writing – original draft, funding acquisition, investigation.

## Funding

There was no funding for this article.

## Disclosure

No Artificial Intelligence (AI) tools were used in the generation, interpretation, analysis, or writing of this manuscript.

## Ethics Statement

This study protocol was approved by the relevant Committee for Ethics of Capital Medical University (Z2023SY051).

## Conflicts of Interest

The authors declare no conflicts of interest.

## Data Availability

The data that support the findings of this study are available from the corresponding author upon reasonable request.
